# First national-scale evaluation of temephos resistance in *Aedes aegypti* in Peru

**DOI:** 10.1186/s13071-022-05310-x

**Published:** 2022-07-11

**Authors:** Miriam Palomino, Jesus Pinto, Pamela Yañez, Anali Cornelio, Luciana Dias, Quesia Amorim, Ademir Jesus Martins, Audrey Lenhart, Jose Bento Pereira Lima

**Affiliations:** 1 Laboratorio de Referencia Nacional de Entomologia, Centro Nacional de Salud Pública, Instituto Nacional de Salud, Lima, Peru; 2 Laboratório de Fisiologia e Controle de Artrópodes Vetores-LAFICAVE, Instituto Oswaldo Cruz (IOC)-Fundação Oswaldo Cruz, Rio de Janeiro, Brazil; 3 Centers for Disease Control and Prevention (CDC), Atlanta, GA USA

**Keywords:** *Aedes aegypti*, Arboviruses, Insecticide resistance, Temephos, Resistance ratio (RR), Vector control

## Abstract

**Background:**

The development of resistance against insecticides in* Aedes aegypti* can lead to operational failures in control programs. Knowledge of the spatial and temporal trends of this resistance is needed to drive effective monitoring campaigns, which in turn provide data on which vector control decision-making should be based.

**Methods:**

Third-stage larvae (L3) from the F1 and F2 generations of 39 Peruvian field populations of *Ae. aegypti* mosquitoes from established laboratory colonies were evaluated for resistance against the organophosphate insecticide temephos. The 39 populations were originally established from eggs collected in the field with ovitraps in eight departments of Peru during 2018 and 2019. Dose–response bioassays, at 11 concentrations of the insecticide, were performed following WHO recommendations.

**Results:**

Of the 39 field populations of *Ae. aegypti* tested for resistance to temephos , 11 showed high levels of resistance (resistance ratio [RR] > 10), 16 showed moderate levels of resistance (defined as RR values between 5 and 10) and only 12 were susceptible (RR < 5). The results segregated the study populations into two geographic groups. Most of the populations in the first geographic group, the coastal region, were resistant to temephos, with three populations (AG, CR and LO) showing RR values > 20 (AG 21.5, CR 23.1, LO 39.4). The populations in the second geographic group, the Amazon jungle and the high jungle, showed moderate levels of resistance, with values ranging between 5.1 (JN) and 7.1 (PU). The exception in this geographic group was the population from PM, which showed a RR value of 28.8 to this insecticide.

**Conclusions:**

The results of this study demonstrate that *Ae. aegypti* populations in Peru present different resistance intensities to temephos, 3 years after temephos use was discontinued. Resistance to this larvicide should continue to be monitored because it is possible that resistance to temephos could decrease in the absence of routine selection pressures.

**Graphical Abstract:**

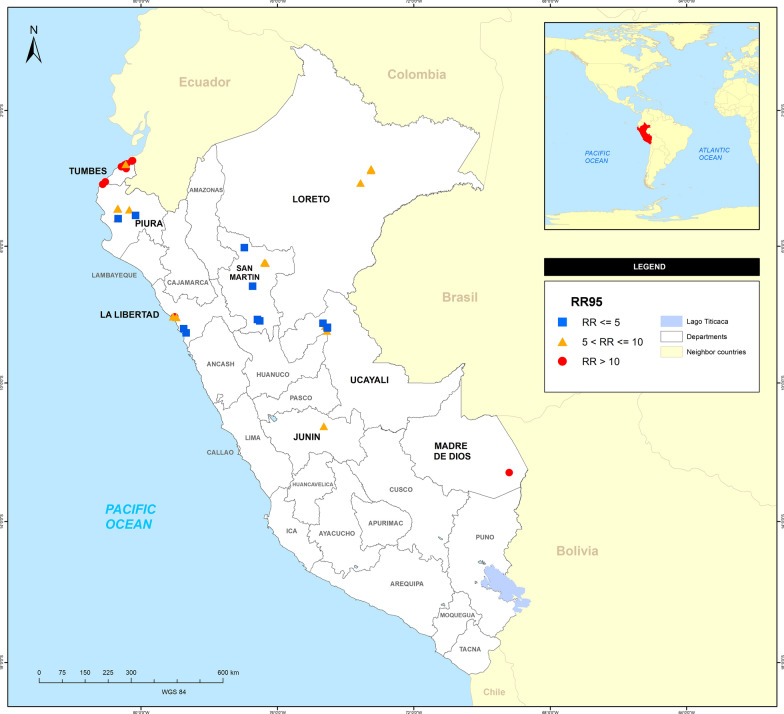

**Supplementary Information:**

The online version contains supplementary material available at 10.1186/s13071-022-05310-x.

## Background

*Aedes aegypti* is the main vector of arthropod-borne viral infections transmitted to humans, which include dengue, yellow fever, chikungunya and Zika [1, 2]. The vector is present in tropical and subtropical regions of Southeast Asia, the Pacific and the Americas, where these viruses also circulate. Within these regions, the vector shows local spatial variations in presence and density that are strongly influenced by rainfall, temperature and degree of urbanization [3]. It has been observed that this mosquito has now adapted to areas at higher altitudes than its traditional range, such as in Cochabamba (Bolivia) at 2550 m a.s.l. [4] and in Bello (Colombia) at 2302 m a.s.l. [5], and that it has colonized places such as Ica (Peru), which has an arid temperate climate with an annual rainfall of only 2 mm [6, 7]. These adaptations have led to a wide dispersal of the vector, which in turn has led to the presence of arboviruses that often follow the same pattern of dispersal.

It is estimated that about 2.5 billion people, representing 40% of the human population worldwide, live in areas at risk of dengue transmission [8] and that 390 million cases of dengue occur per year in tropical and subtropical areas [3]. In addition, in the last 5 years, *Ae. aegypti* has been responsible for the spread of chikungunya and Zika to regions of the Americas, placing a significant burden on healthcare systems and also causing social and economic disruption [9]. While *Ae. albopictus* is widely distributed in the Americas (present in 21 of 44 countries), it has not been detected in Peru. However, Peru shares borders with countries reporting widespread distribution of *Ae. albopictus*, such as Brazil, Colombia, Bolivia and Ecuador (where *Ae. albopictus* was first detected in 2017) [10].

In Peru, the vector of dengue, chikungunya and Zika is *Ae. aegypti*. *Aedes aegypti* was eradicated locally in 1958, but it subsequently recolonized the country in 1984 [11]. The first outbreak of dengue occurred 6 years later, attributed to dengue virus serotype 1 (DENV-1) [12]. Low-incidence outbreaks occurred thereafter up to 2001, when a major outbreak occurred, with 23,304 cases. This outbreak confirmed the circulation of the four serotypes of DENV [13]. Significant outbreaks occurred between 2017 and 2020, with 68,290 cases and 52,826 cases, respectively [14]. The first reported cases of chikungunya and Zika occurred in 2015 and 2016, respectively [15, 16].

In Peru, *Aedes*-borne arboviruses are present in three ecological regions: the coast, the Amazon jungle and the Andes Mountains [17, 18]. The latter region (Andes Mountains) presents such a diversity of altitudes that is differentiated into two areas: the Andean highlands (> 2300 m a.s.l.) and high jungle (400—1400 m a.s.l.; located on the eastern flank of the Andes) [19]. *Aedes*-borne arboviruses only affect areas of lower altitude. *Aedes aegypti* are widely distributed in 21 of Peru’s 24 departments and the constitutional province of Callao, and have been identified in 527 districts [20], where approximately 22 million people live, putting 70.4% of the Peruvian population at risk of contracting arboviruses transmitted by this vector.

The main dengue control strategy implemented in Peru until 2016 was focal treatment of larval habitats with the organophosphate (OP) insecticide temephos due to its easy dosage, application and acceptability by the community. For the same reasons, the adulticides used in the 1990s were the OPs fenitrothion and malathion [21]. Since the beginning of this century, pyrethroid (PY) insecticides have also been used (cyfluthrin, deltamethrin, alpha-cypermethrin and cypermethrin) [17, 22], applied using thermal and cold fog equipment that can be manually carried or mounted on trucks. However, in 2015, Pinto et al. [23] detected resistance against PYs in field-caught *Ae. aegypti* populations in association with knockdown resistance (*kdr*) mutations (Phe1534Cys, Val1016Ile). This led the Peruvian Ministry of Health (MINSA) to implement a control strategy in which PYs were rotated with the OP malathion. For larval control, the OP temephos was switched with the insect growth regulator (IGR) pyriproxyfen because temephos shares the same mode of action as the OP malathion. This switch away from using temephos, however, was made without any knowledge of the susceptibility status of *Ae. aegypti* populations to this insecticide.

Surveillance of insecticide resistance in arthropod vectors in Peru is an activity that falls under the Instituto Nacional de Salud (INS; National Institute of Health) and Regional Reference Laboratories (LRRs) which, in the context of a decentralized healthcare system, requires coordinated work. However, many of the LRRs do not have entomology laboratories or insectaries and thus do not have the facilities or knowledge to routinely perform the various tasks/responsibilities placed on them [22, 24]. This situation results in a weakening of the vector surveillance and control programs, despite ongoing increases in the numbers of cases of *Aedes*-borne arboviruses. The continuous use of insecticides results in selective pressures that in turn drive physiological and/or behavioral adaptation, a phenomenon known as resistance [25]. Insecticide resistance can lead to operational failures, ultimately requiring rotations of insecticides with different modes of action or mosaic treatments to manage insecticide resistance [26]. Ideally, these strategies should be carried out preemptively to preserve insecticide efficacy, but also reactively to mitigate or reverse resistance [25].

The aim of the present study was to determine the levels of resistance to temephos in 39 Peruvian field populations of *Ae. aegypti* from the three ecological regions of Peru after having used temephos for > 25 years, following an interruption in its use of 3 years.

## Methods

### Sampling and study area

**Fig. 1:**
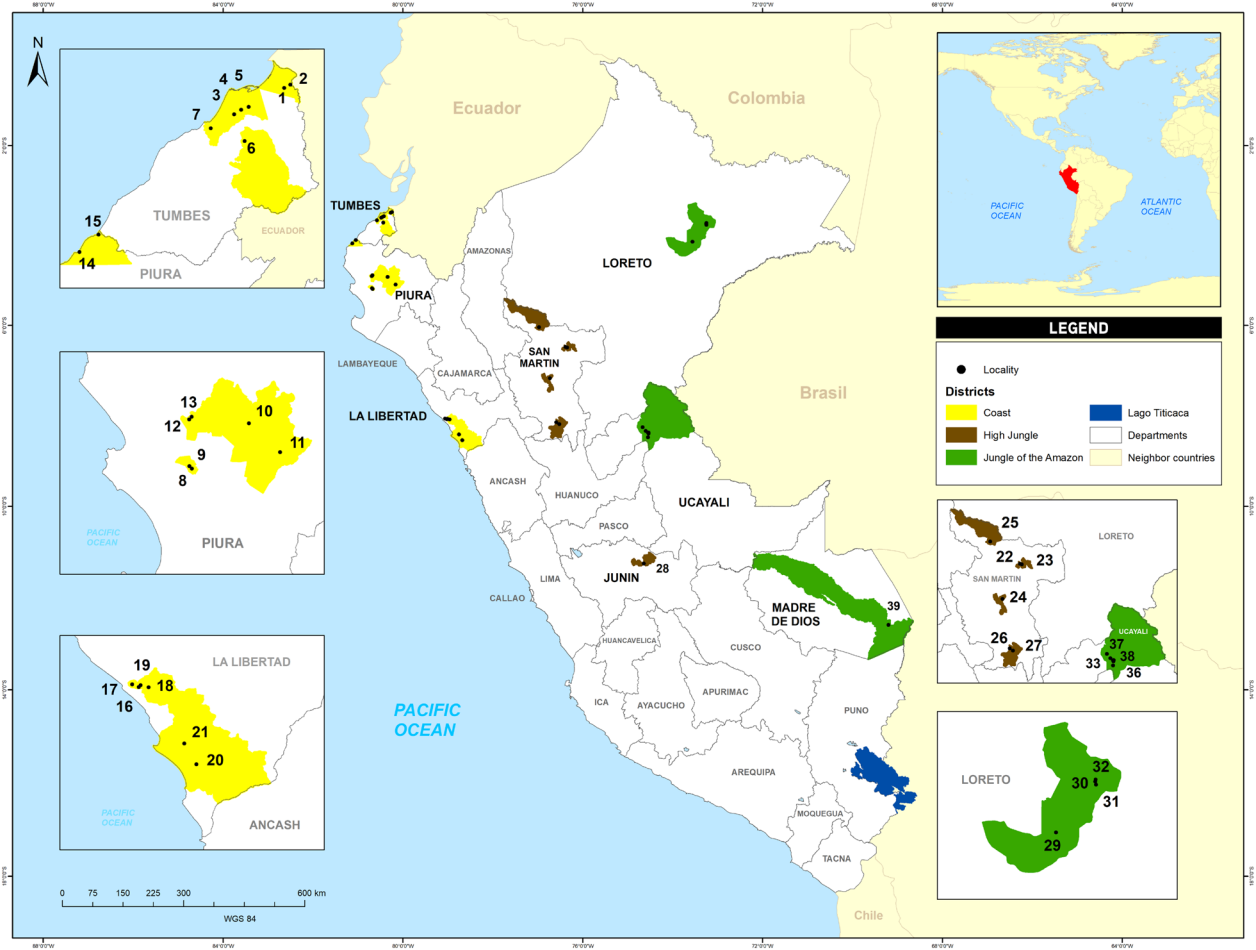
Map of the selected localities in the three ecological regions of Peru where the 39 populations of *Aedes aegypti* originated.* 1* Zarumilla (ZA),* 2* Aguas Verdes (AG),* 3* Corrales (CR),* 4* Pampa Grande (PG),* 5* Sagaro (SA),* 6* Cabuyal (CB),* 7* La Cruz (CZ),* 8* San José (JO),* 9* Micaela Bastidas (MI),* 10* Tambogrande (TA),* 11* Chulucanas (CH),* 12* Bellavista (BE),* 13* Comunidad Saludable (CS),* 14* Los Órganos (LO),* 15* Máncora (MA),* 16* El Porvenir (PO),* 17* La Esperanza (ES),* 18* Laredo (LA),* 19* Florencia de Mora (FL),* 20* Chao (CA),* 21* Virú (VI),* 22* Morales (MR),* 23* Banda de Shilcayo (BS),* 24* Juanjui (JJ),* 25* Moyobamba (MO),* 26* Nuevo Bambamarca (BM),* 27* Tocache (TO),* 28* Satipo (ST),* 29* San Juan Bautista (JN),* 30* Iquitos (IQ),* 31* Belén (BN),* 32* Punchana (PU),* 33* Manantay (MY),* 34* San Fernando (FE),* 35* Callería (PL),* 36* José Carlos Mariátegui (PA),* 37* Yarinacocha (YA),* 38* San José Yarinacocha (SY),* 39* Puerto Maldonado (PM)

The mosquito population tested comprised the F1 or F2 generations of *Ae. aegypti* from colonies maintained at INS that were established from eggs collected in 39 localities in the Peruvian departments of Tumbes, Piura, La Libertad, San Martín, Loreto, Ucayali, Junin and Madre de Dios during April 2018 and January 2019 (Fig. 1). The colonies were obtained from eggs collected with ovitraps, which were distributed every 200 linear meters covering the urban area of the locality, according to the parameters established by MINSA [27]. The ovitraps were installed in intra- and peri-domestic areas and contained a strip of paper towel as oviposition substrate [27, 28] and 10% hay infusion as attractant [29]. The localities sampled are located in three ecological regions showing distinct weather patterns [6], variations in the incidence of dengue [17, 18] and *Ae. aegypti* populations with differing insecticide resistance profiles [23].

### Mosquito collection and laboratory rearing

The paper strips containing eggs were left to dry for 1 week and then soaked in water to hatch the F0 generation [30]. The F1 and F2 generations were subsequently established in the insectary of the Laboratorio de Referencia National de Entomología (LRNE), INS, Lima, Peru (Table 1). Humidity and temperature conditions in the insectary were maintained at 70 ± 10% relative humidify and 26 ± 2 °C, respectively. *Aedes aegypti* of the susceptible Rockefeller reference strain were used as a control for the resistance tests [31].

**Table 1:** *Aedes aegypti* populations used in all tests

Locality/population	Code	Collection date	Number of eggs collected	Generation evaluated
Aguas Verdes	AG	April 2018	1148	F1
Zarumilla	ZA	April 2018	1025	F1
Sagaro	SA	April 2018	748	F1
Pampa Grande	PG	April 2018	2448	F1
Corrales	CR	April 2018	833	F1
Cabuyal	CB	April, 2018	233	F1
La Cruz	CZ	April 2018	187	F1
Mancora	MA	April–May 2018	2051	F1
Los Órganos	LO	April–May 2018	1573	F1
Bellavista	BE	April–May 2018	1992	F1
Comunidad Saludable	CS	April–May 2018	515	F1
Micaela Bastidas	MI	April–May 2018	468	F1
San José	JO	April–May 2018	1062	F1
Tambogrande	TA	April 2018	1360	F1
Chulucanas	CH	April–May 2018	2398	F1
La Esperanza	ES	April 2018	4580	F1
El Porvenir	PO	April 2018	3023	F1
Florencia de Mora	FL	April 2018	1673	F1
Laredo	LA	May 2018	2442	F1
Chao	CA	April 2018	1507	F1
Virú	VI	April 2018	781	F1
Moyobamba	MO	November–December 2018	n.a	F2
Morales	MR	November 2018	900	F1
Banda de Shilcayo	BS	November 2018	n.a	F2
Juanjui	JJ	December 2018	1846	F1
Nuevo Bambamarca	BM	November 2018	n.a	F2
Tocache	TO	November 2018	n.a	F2
Satipo	ST	September 2018	1216	F1
Iquitos	IQ	June 2018	6651	F1
Punchana	PU	June 2018	733	F1
Belén	BN	June 2018	775	F1
San Juan Bautista	JN	June 2018	1876	F1
San José	SY	August 2018	324	F1
Yarinacocha	YA	August 2018	2342	F1
Callería	PL	August 2018	2123	F1
José Carlos Mariátegui	PA	August 2018	1487	F1
Manantay	MY	August 2018	1391	F1
San Fernando	FE	August 2018	562	F1
Puerto Maldonado	PM	January 2019	891	F1

*n.a. *Data not available

### Larvicide susceptibility testing

Dose–response susceptibility tests were performed with temephos following WHO recommendations [32]. The larvae were exposed to a wide range of concentrations of the insecticide with the aim to evaluate larvicidal activity and thereby determine the 50% and 95% lethal concentration values (LC_50_ and LC_95_) for each study population. Four replicates per concentration, and 11 concentrations were evaluated with 20 third-stage larvae (L3) in 100 ml of insecticide solution per replicate. Insecticide solutions were prepared with ethanol solvent and temephos active ingredient (Chem Service, West Chester, PA, USA), using a concentration range of 0.004 to 0.324 mg/ml for the field populations, and 0.002 to 0.012 mg/ml for the control Rockefeller strain. Simultaneously, a control group with four replicates exposed only to 600 µl of ethanol solvent was evaluated [32]. Each test was carried out on three different days to ensure the reproducibility of the method and consistency of the results [32, 33].

### Data analysis

The values for LC_50_ and LC_95_ were calculated from a log dosage-probit mortality regression line using probit analysis (Polo-PC statistics package [34]) for each population. Resistance ratios (RRs; i.e. LC of field population/LC of susceptible population) were also calculated to define the intensity of resistance in the field populations. Specifically, Eq. 1 was used to calculate the RR_50_ (where *i* = 50) and the RR_95_ (where *i* = 95 as shown::

1 $${\mathrm{RR}}_{{\varvec{i}}}=\frac{{\mathrm{LC}}_{{\varvec{i}}\left(\mathrm{field}\right)}}{{LC}_{{\varvec{i}}\left(\mathrm{Rockefeller \, Lineage}\right)}}$$

The population was considered to be susceptible when the RR < 5; when the RR was between 5 and 10, the population was considered to have moderate resistance; and when the RR ≥ 10, the population was to be considered highly resistant.

## Results

Third-stage larvae from the F1 and F2 generations of 39 Peruvian populations of *Ae. aegypti* mosquitoes from localities with different incidences of arboviruses and located in three ecological regions of Peru were tested. A total of 37,440 L3 were tested across all bioassays.

**Fig. 2:**
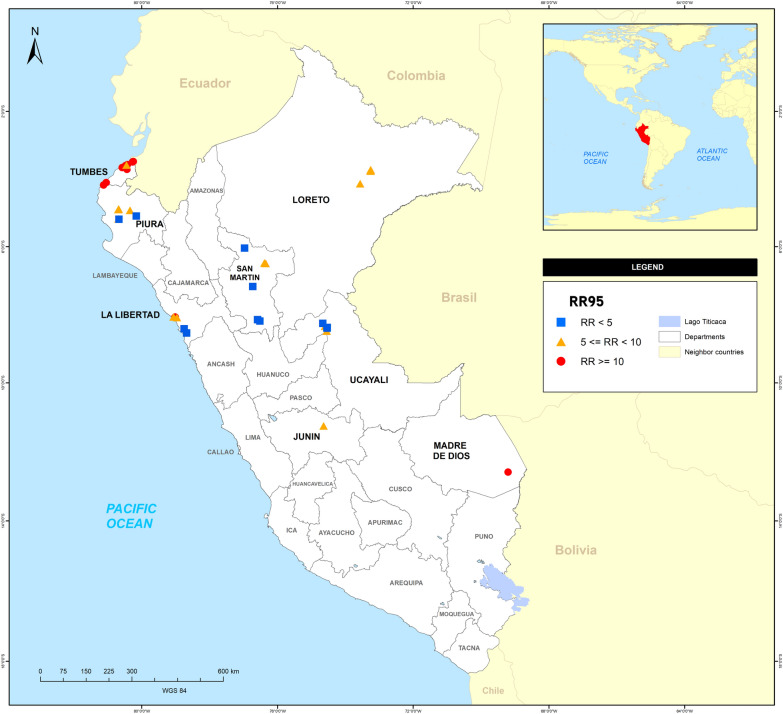
Temephos resistance ratios in Peruvian populations of *Ae. aegypti* 2018–2019. Abbreviations: RR Resistance ratio (see Eq. 1)

Table 2 and Fig. 2 show the values of the LC_50_ and LC_95_, the resistance ratios (RR_50_ and RR_95_) and the slope of the probit regression lines for the insecticide temephos in the 39 populations of *Ae. aegypti*. The differences between the RR_95_ of all populations were analyzed according to the criteria of Mazzarri and Georghiou [35] and Sá et al. [33].The results showed that the three regions could be grouped into two groups: coastal and jungle (the latter including the Amazon and high jungles). Most of the population in the coastal group showed high levels of resistance to temephos (RR > 10), with three populations showing values > 20 (AG 21.5, CR 23.1, LO 39.4; for abbreviations of localities/populations, see Table 1 and Fig. 1). Some populations in th coastal group (PG, MI, TA, BE, CS, ES and LA) showed moderate levels of resistance (5 ≤ RR < 10) and only four populations (JO, CH, CA and VI) were considered to be susceptible (RR < 5). In the jungle group, most of the populations showed moderate levels of resistance (5 ≤ RR < 10), with RR values ranging from 5.1 in JN to 7.1 in PU, while some populations were considered to be susceptible (RR < 5), with values ranging from 2.2 in MO to 4.9 in YA and PA; the notable exception in the jungle group was the PM population, which was considered to be highly resistant (RR = 28.8).

**Table 2:** Temephos susceptibility profiles of Peruvian *Aedes aegypti* populations showing slope values, lethal concentration values and resistance ratios

Department	Lineage/population	Code	*F*	*N*	Slope	LC (95% CI)				RR^a^	
						LC50		LC95		RR_50_	RR_95_
	Rockefeller (control strain)			2880	5.67	0.005	(0.004_0.006)	0.01	(0.009_0.011)	1.0	1.0
Tumbes	Aguas Verdes	AG	F1	2880	2.50	0.047	(0.045_0.050)	0.215	(0.193_0.245)	**9.4**	***21.5***
	Zarumilla	ZA	F1	2880	3.22	0.036	(0.034_0.037)	0.116	(0.108_0.127)	**7.2**	***11.6***
	Sagaro	SA	F1	2880	3.65	0.039	(0.038_0.041)	0.110	(0.103_0.119)	**7.8**	***11.0***
	Pampa Grande	PG	F1	2880	3.72	0.032	(0.031_0.033)	0.089	(0.083_0.095)	**6.4**	**8.9**
	Corrales	CR	F1	2880	2.28	0.044	(0.041_0.046)	0.231	(0.201_0.273)	**8.8**	***23.1***
	Cabuyal	CB	F1	2880	2.42	0.028	(0.026_0.030)	0.135	(0.122_0.152)	**5.6**	***13.5***
	La Cruz	CZ	F1	2880	3.74	0.056	(0.054_0.058)	0.154	(0.145_0.166)	***11.2***	***15.4***
Piura	Mancora	MA	F1	2880	3.7	0.071	(0.069_0.074)	0.199	(0.181_0.222)	***14.2***	***19.9***
	Los Organos	LO	F1	2880	3.01	0.111	(0.090_0.136)	0.394	(0.321_0.484)	***22.2***	***39.4***
	Bellavista	BE	F1	2880	3.05	0.025	(0.024_0.026)	0.086	(0.080_0.094)	**5.0**	**8.6**
	Comunidad Saludable	CS	F1	2880	2.75	0.024	(0.022_0.026)	0.094	(0.087_0.103)	*4.8*	**9.4**
	Micaela Bastidas	MI	F1	2880	3.36	0.020	(0.018_0.021)	0.060	(0.057_0.065)	*4.0*	**6.0**
	San Jose	JO	F1	2880	3.88	0.016	(0.015_0.016)	0.041	(0.039_0.045)	*3.2*	*4.1*
	Tambogrande	TA	F1	2880	4.58	0.025	(0.024_0.026)	0.057	(0.054_0.060)	**5.0**	**5.7**
	Chulucanas	CH	F1	2880	4.02	0.015	(0.014_0.015)	0.038	(0.036_0.041)	*3.0*	*3.8*
La Libertad	La Esperanza	ES	F1	2880	2.54	0.018	(0.016_0.019)	0.078	(0.071_0.087)	*3.6*	**7.8**
	El Porvenir	PO	F1	2880	2.59	0.025	(0.023_0.026)	0.107	(0.097_0.119)	**5.0**	***10.7***
	Florencia de Mora	FL	F1	2880	2.81	0.029	(0.028_0.031)	0.113	(0.103_0.125)	**5.8**	***11.3***
	Laredo	LA	F1	2880	3.37	0.021	(0.020_0.022)	0.066	(0.061_0.071)	*4.2*	**6.6**
	Chao	CA	F1	2880	7.18	0.010	(0.010_0.010)	0.021	(0.020_0.023)	*2.0*	*2.1*
	Viru	VI	F1	2880	5.63	0.011	(0.011_0.012)	0.022	(0.022_0.024)	*2.2*	*2.2*
San Martín	Moyobamba	MO	F2	2880	4.39	0.009	(0.009_0.010)	0.022	(0.021_0.024)	*1.8*	*2.2*
	Morales	MR	F1	2880	4.00	0.021	(0.021_0.022)	0.055	(0.052_0.059)	*4.2*	**5.5**
	Banda de Shilcayo	BS	F2	2880	4.33	0.023	(0.022_0.024)	0.055	(0.053_0.059)	*4.6*	**5.5**
	Juanjui	JJ	F1	2880	3.65	0.010	(0.009_0.010)	0.042	(0.039_0.047)	*2.0*	*4.2*
	Nuevo Bambamarca	BM	F2	2880	4.85	0.011	(0.010_0.011)	0.023	(0.022_0.025)	*2.2*	*2.3*
	Tocache	TO	F2	2880	4.14	0.010	(0.009_0.010)	0.024	(0.023_0.026)	*2.0*	*2.4*
Junin	Satipo	ST	F1	2880	3.45	0.019	(0.018_0.020)	0.058	(0.054_0.062)	*3.8*	**5.8**
Loreto	Iquitos	IQ	F1	2880	4.02	0.026	(0.025_0.027)	0.067	(0.063_0.071)	**5.2**	**6.7**
	Punchana	PU	F1	2880	4.73	0.032	(0.031_0.033)	0.071	(0.068_0.075)	**6.4**	**7.1**
	Belen	BN	F1	2880	4.36	0.023	(0.022_0.024)	0.054	(0.052_0.057)	*4.6*	**5.4**
	San Juan Bautista	JN	F1	2880	4.18	0.021	(0.020_0.022)	0.051	(0.048_0.054)	*4.2*	**5.1**
Ucayali	San Jose-Yarinacocha	SY	F1	2880	3.89	0.017	(0.017_0.018)	0.069	(0.063_0.076)	*3.4*	**6.9**
	Yarinacocha	YA	F1	2880	4.21	0.02	(0.019_0.020)	0.049	(0.046_0.052)	*4.0*	*4.9*
	Pucallpa 1	PL	F1	2880	4.02	0.015	(0.014_0.015)	0.038	(0.036_0.041)	*3.0*	*3.8*
	Pucallpa 2	PA	F1	2880	3.83	0.018	(0.018_0.019)	0.049	(0.046_0.053)	*3.6*	*4.9*
	Manantay	MY	F1	2880	3.58	0.018	(0.017_0.019)	0.052	(0.048_0.056)	*3.6*	**5.2**
	San Fernando	FE	F1	2880	5.07	0.015	(0.014_0.015)	0.031	(0.030_0.033)	*3.0*	*3.1*
Madre de Dios	Puerto Maldonado	PM	F1	2880	2.11	0.048	(0.045_0.051)	0.288	(0.243_0.355)	**9.6**	***28.8***

*CI* Confidence interval, *LC50/95* lethal concentration of temephos that killed 50 and 95% of the test population, respectively,* RR* resistance ratio (for* RR*_*50*_/*RR*_*95*_ definition, see Eq. 1 in text)

^a^Values in italics: RR < 5. Values in bold: 5 ≤ RR < 10; values in bold italics: RR ≥ 10

However, the RRs of the populations were heterogeneous, even within the same department. In Tumbes, for example, most populations were highly resistant to temephos (RR > 10), except for the PG population (RR = 8.9) which showed moderate resistance to this insecticide. Piura, in comparison, had two populations (MA and LO) that exhibited high RR values (RR > 19), but also had two susceptible populations with low values (RR < 5). In La Libertad, the RRs varied between 2.1 in CA and 11.3 in FL, showing that the mosquito populations in this department also showed heterogeneous resistance RRs; however, there were also two populations in which the lowest RRs were detected (RR < 2.5). The departments of San Martin, Junín, Loreto and Ucayali were more homogeneous, with RR values ranging from 2.2 in MO to 7.1 in PU. However, despite the generally lower RR values in the jungle areas, in the jungle department of Madre de Dios, the PM population showed a RR value of 28.8. However, the RRs of the populations were heterogenous even within in the same locality. Populations AG, ZA, SA, PG, CR, CB, PO, FL and PM had moderate levels of resistance (5 ≤ RR < 10) in the RR_50_ but showed high resistance to temephos (RR > 10) in the RR_95_, and populations CS, MI, ES, LA, MR, BS, ST, BN, JN, SY and MY) had low values in the RR_50_ but showed moderate resistance in the RR_95_. Populations CZ, MA and LO were more homogeneous due to their high resistance to temephos, with high values of RR_50_ (11.2, 14.2 and 22.2, respectively) and RR_95_ (15.4, 19.9 and 39.4, respectively). Some populations (PG, BE, TA, IQ and PU) showed moderate levels of resistance (5 ≤ RR < 10), and 12 populations (JO, CH, CA, VI, MO, JJ, BM, TO, YA, PL, PA and FE) were considered to be more susceptible (RR < 5) in both the RR_50_ and RR_95_. In general, jungle localities were more homogeneous.

**Fig. 3:**
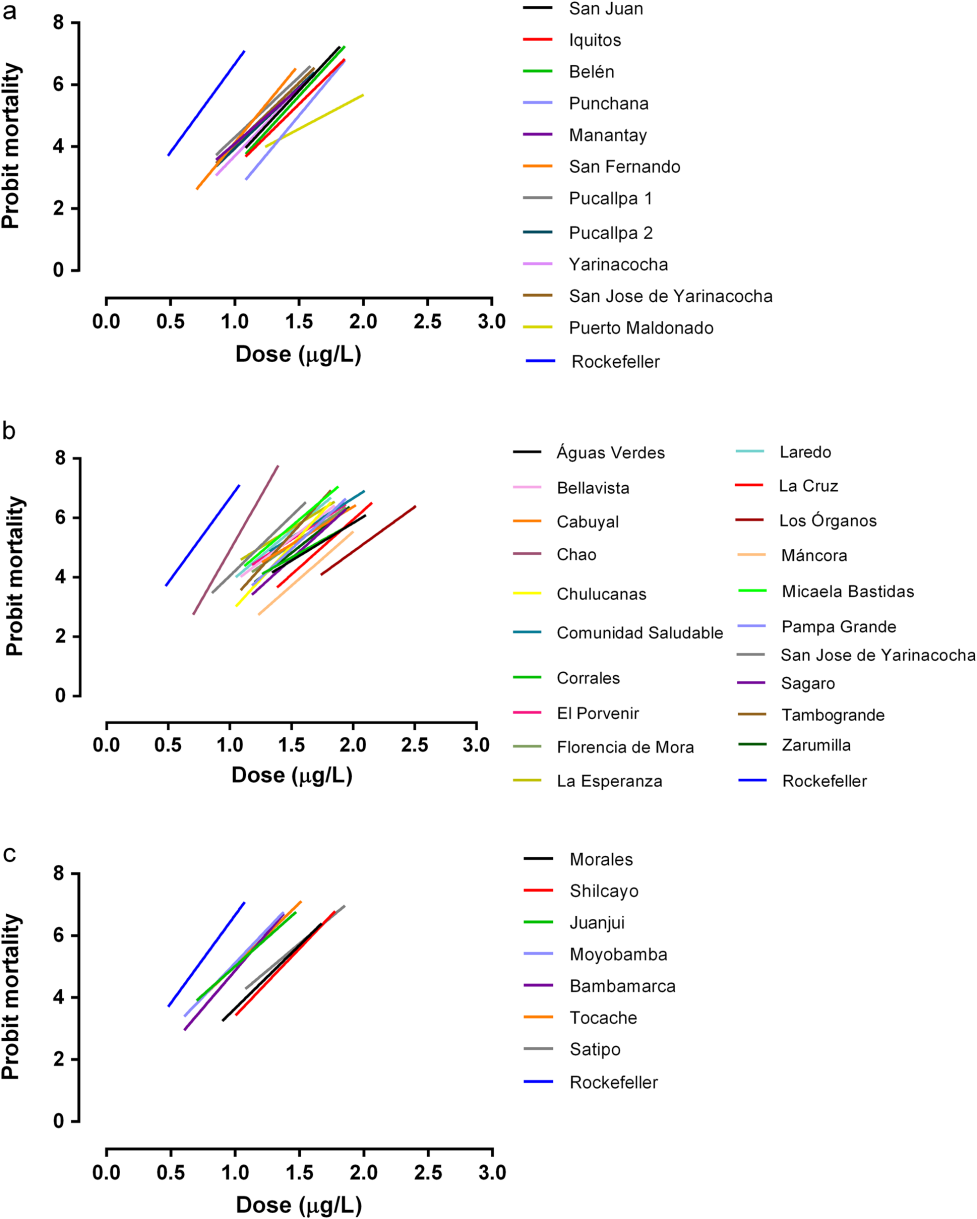
Linear regression of *Ae. aegypti* mortality after exposure to the organophosphate temephos in Peruvian populations compared to the susceptible Rockefeller strain (in blue). **a** The Amazon jungle, **b** Coast, **c** High jungle

 The slope values of the probit regression lines of the field populations were lower than those obtained with the Rockefeller lineage, except in the CA population (slope = 7.18) (Table 2; Fig. 3). This result confirms the heterogeneity of temephos resistance in the field strains in relation to the reference strain.

## Discussion

As part of a national strategic plan to eliminate dengue, chikungunya and Zika, a national survey to ascertain the susceptibility of *Ae. aegypti* to temephos was undertaken. The Peruvian INS, in coordination with Regional Health Directorates, surveyed 39 dengue endemic localities from April 2018 to January 2019. Overall, Peruvian populations of *Ae. aegypti* showed variable levels of resistance to temephos. Based on the results, these populations were clustered into two groups: (i) coastal populations with moderate to high levels of resistance to temephos, and (ii) jungle populations, which showed a moderate level of resistance to temephos, and some susceptibility, except for the Puerto Maldonado population which showed a very high level of resistance. Interestingly, from 2015 to 2017, the dengue cases reported in the coastal departments accounted for between 54 and 86% of the total number of dengue cases reported in Peru, while from 2018 to 2021, dengue cases reported in the jungle regions accounted for between 55 and 87% of the total number of dengue cases reported [36, 37] (Additional file 1: Table S1).

Higher levels of resistance to temephos were detected in coastal populations than in jungle populations, despite the northern coast having been recolonized by *Ae. aegypti* 10 years later than the Peruvian Amazon [38, 39]. The reasons underlying this difference in susceptibility are likely diverse. One possible explanation is based on the notion that *Ae. aegypti* entered the country along its border with Ecuador [11]. It is known that *Ae. aegypti* populations in Ecuador have variable levels of resistance to temephos, with populations from Huaquillas (bordering Peru) and Arenas showing susceptibility, while the populations from San Lorenzo and Nueva Loja show high levels of resistance. This variability could be due to variations in the intensity and frequency of temephos use across Ecuador [40]. Another possible explanation is climate differences. The departments along the northern coast of Peru were the most affected by the El Niño climatic phenomenon (1997–1998), with heavy rains that caused an increase in malaria and dengue cases [41, 42]. This situation led to the establishment of prevention and control activities, including insecticide space spraying and indoor residual spraying, mapping and treatment of larval habitats (including chemical control with temephos) and campaigns to eliminate potential larval habitats. These activities were carried out periodically in 333 localities in the departments of Tumbes, Piura, Lambayeque and La Libertad, with the goal of eliminating larval habitats in urban, peri-urban and rural areas. In addition, the urban population on the coast is growing, but there is a lack of planning and organization and thus insufficient basic sanitation facilities due to, among other reasons, constant migration from other regions due to violence, lack of job opportunities, education and access to technological resources, as well as poverty [42]. This rapid, unplanned urbanization favors the introduction and establishment of *Ae. aegypti*. However, the west coast of Peru is a long desert strip interrupted by valleys, and the main characteristic of the region is a scarcity of rainfall [6], which should serve as a limiting factor for the transmission of arboviruses in this region, with the one important exception being the north coast, which experiences high temperatures and rainfall in the summer due to its proximity to the equator [18]. Overall, therefore, the potential benefit of the mainly arid climate is partially or totally negated by the presence of larval habitats inside homes as a consequence of poor water storage facilities due to an inadequate piped water supply in urban centers [17, 26]. Evidence of this can be seen in the types of larval habitats that predominate in and around homes, including water storage drums, cylinders, wells and flower vases [7]. As a result, the lack of access to and availability of water, as well as the poor quality of water, create conditions for the proliferation of vectors and transmission of arboviruses [43], as recognized by the Ministry of Health of Peru, which reported in 2016 that inadequate access to water was associated with 41.2% of cases of dengue [44].

The ecosytems of the Peruvian Amazon differ from those on the coast. These jungle areas typically include humid and rainy tropical forests [6]. In this region, due to the constant rainfall, there is a greater abundance of potential larval habitats, especially those known as “los inservibles” (discarded objects, passively filled with rainwater). Unlike along the coast, these larval habitats are typically peri-domestic and are not used intentionally to hold water [45]. In contrast to the urbanization along the coast, the jungle is the least populated region of the country, accounting for only 10% of the national population, and the infrastructure of Amazonian villages often reflects inadequate access to basic services, education and health [46]. The city of Puerto Maldonado is the capital of the Amazonian department of Madre de Dios, located in the southeastern part of the country. The *Ae. aegypti* population in this city was the only one in the Peruvian Amazon that presented a high level of resistance to temephos. The vector was introduced into this city in 1999 [47], 15 years after it first being recorded in the Peruvian jungle in 1984 [11]. Dengue cases were sporadic in the period 2000–2016, with only one major outbreak (2952 recorded cases) reported in 2010 [48]. This low burden of dengue is supported by the findings of Salmón-Mulanovich et al. [49], who found low seroprevalence to DENV in a retrospective study conducted in Puerto Maldonado in 2018. This low disease burden suggests that vector control activities were not routine or intense and that the local *Ae. aegypti* population has not experienced strong selection pressure from the larvicide temephos. The explanation for the high levels of temephos resistance detected in the present study could be due to the vector having spread across the border from Brazil and Bolivia. On the Brazilian side, the *Ae. aegypti* population from Rio Branco (Acre State) is highly resistant to temephos [50] and similarly, on the Bolivian side, the *Ae. aegypti* population in the border city of Cobija (department of Pando) shows moderate levels of resistance to temephos [51].

It is also important to consider that the use of pesticides in the agricultural sector in Peru is intense at the national level, with the most widely used insecticides being OPs [52]. In a historical context, organochlorine (OC) pesticides were used between 1940 and 1950 (dichlorodiphenyltrichloroethane [DDT], benzene hexachloride [BHC] and toxaphene), followed by the introduction of the OPs parathion and methamidophos in 1950 and their use for several decades, with parathion used until 2005 [52] and methamidophos until 2018–2019 [53]. In the 2000s, pyrethroids (deltamethrin, cypermethrin and alpha-cypermethrin) and carbamates [CA] (carbofuran, methomyl, carbosulfan and carbaryl) were introduced [52, 53]. A study conducted in 2012 reported that 43% of farmers preferred to use OPs because they had a broad spectrum of action (contact, ingestion and fumigant effects) [54]. It is important to note that these pesticides were widely used on cotton, corn and potato crops in the northern and central coastal valleys, as well as in the Andes Mountains. Another important consideration is the illegal trade in pesticides through smuggling (mainly along the northern border), street sales and adulteration and counterfeiting of products, especially in the northern coast, central and southern parts of the Andes Mountains [55]. The continuous use of pesticides in agriculture has led to pest resistance to OCs and OPs due to misuse of pesticides and lack of pesticide management [52]. The agricultural sector is using the same classes of insecticides that are being used for public health, so mosquitoes and other non-target insects may experience selection pressure from insecticides used in agriculture, resulting in the selection of populations that exhibit resistance to multiple insecticides [56].

The results of the present study demonstrate that Peruvian *Ae. aegypti* populations show diverse levels of resistance to the OP temephos, and are consistent with resistance patterns observed in other field populations that have been subjected to intense selection pressure from temephos [57]. In Peru, temephos was used for > 25 years; it is thus not surprising that resistance to this insecticide has reached very high levels in some areas. However, it is not possible to estimate or quantify the evolution of this resistance due to the lack of baseline information; the absence of data from Peru is also found in the review of resistance prepared by Moyes et al. [58]. Resistance to temephos has been reported worldwide, with high levels of resistance reported in Tamil Nadu (India) [59], Caldas (Colombia) [60], Pernambuco (Brazil) [61], Martinique [62] and Bahia (Brazil) [63]; moderate levels of resistance reported in Tocantins (Brazil) [33], Laos (Asia) [64], Paraná (Brazil) [65], Quindío (Colombia) [66], Delhi (India) [67], Sao Paulo and Northeast Region (Brazil) [68]; and susceptibility reported in Malaysia [69], Santiago Island (Cape Verde) [70] and Phitsanulok province in Thailand [56].

Chemical control of *Ae. aegypti* with temephos in Peru was continuous until 2015 when it was replaced by pyriproxyfen [71]; this replacement could explain the moderate and low levels of resistance found in the populations of Pampa Grande, Bellavista and Comunidad Saludable, among others. This finding suggests that resistance to temephos is unstable in the absence of continuous selection pressure. The notion of unstable resistance is supported by the findings of various researchers. Wirth and Georghiou [57] who detected significant decreases in the levels of resistance to temephos in the British Virgin Islands (Tortola) following an interruption of > 10 years in its use, from a RR of 46.8 in 1985 [24] to a RR of 12.1 in 1992–93 [72] and then to a RR of 6.3 in 1995–96 [73]. Similarly, in Colombia (Caldas), Conde et al. [60] observed a reduction in the level of resistance to temephos at > 4 years after discontinuation of its use, from RRs of 13.27 and 11.48 in 2007 to RRs of 4.75 and 5.61 in 2011. Similarly, in Brazil (Juazeiro do Norte) [74], a decrease in resistance to temephos was observed, from a RR of 10.4 to a RR of 7.2, 7 years after this larvicide was replaced by *Bacillus thuringiensis israelensis* (Bti). Also in Brazil, Rahman et al. [75] observed a reduction in the level of resistance to temephos in Rio de Janeiro, in the municipality of Campos dos Goytacazes, 15 years after discontinuationof its use, from a RR of 7.8 in 2001 to a RR of 2.6 in 2016. These authors also detected a significant decrease in the levels of resistance to temephos in the municipality of Itaperona, from a RR of 25.6 in 2011 to a RR of 7.3 in 2016, after this larvicide was substituted by IGR [75]. Consequently, rotating to a new insecticide with a different mode of action could be advantageous in terms of temephos resistance management. The WHO recommends the following compounds as alternative larvicides: Bti, diflubenzuron, methoprene, novaluron, pyriproxyfen and spinosad.

Following the observed resistance of Peruvian *Ae. aegypti* to pyrethroids, the OP malathion is being reintroduced [71]. It is uncertain how long this insecticide will remain effective if resistance to temephos has already been demonstrated or if there is cross-resistance between temephos and malathion. Wirth and Georghiou [57] suggested that resistance to malathion did not increase significantly under selection pressure with temephos and that adulticides exerted lower selection pressure than larvicides [28]. If this is indeed the case, it is reasonable to believe that malathion may still be effective in Peru, which is important considering the few chemical options available in Peru for vector control.

An important limitation to this study was the lack of additional evaluations that would allow us to better understand the evolution of resistance to temephos, as well as the characterization of the observed resistance by molecular and enzymatic methods. On this last point, Rodriguez et al. [76], in a study with Latin American populations, found that a Peruvian *Ae. aegypti* population presented variations in the intensity of resistance to different OPs (temephos, RR = 30; malathion, 1.5; fenthion, 6.6; pirimiphos-methyl, 10; fenitrothion, 1.1; chlorpyrifos, 4.3), with elevated activity of esterases related to resistance to temephos, while mono-oxygenases were associated with resistance to pirimiphos-methyl and chlorpyrifos.

## Conclusions

The results of this study demonstrate that *Ae. aegypti* populations in Peru have different levels of resistance to temephos, even after its widespread use was suspended, spanning the spectrum from susceptible to high levels of resistance. It is necessary to continue monitoring resistance to this larvicide to understand if in the absence of its widespread use, susceptibility may be recovered in the future. The implementation of insecticide resistance management strategies is important to preserve the efficacy of insecticides used in public health programs, and it is necessary to carry out vector control with multisectoral components that can improve the effectiveness of the *Ae. aegypti* control program in Peru.

## Supplementary Information

**Additional file 1: Table S1.** Number of cases of dengue according to departments and geographic regions, Peru 2015–2021.

## Data Availability

All data generated or analyzed during this study are included in this published article.

## References

[1] Hamid PH, Ninditya VI, Ghiffari A, Taubert A, Hermosilla C (2020). The V1016G mutation of the voltage-gated sodium channel (VGSC) gene contributes to the insecticide resistance of *Aedes aegypti* from Makassar, Indonesia. Parasitol Res.

[2] Fontana JD, Ferreira RL, Zuccolotto T, de BorbaDallagassa C, Wielewski LP, Chalcoski BMS (2020). Accelerating the morphogenetic cycle of the viral vector *Aedes aegypti* larvae for faster larvicidal bioassays. Biomed Res Int.

[3] Bhatt S, Gething PW, Brady OJ, Messina JP, Farlow AW, Moyes CL (2013). The global distribution and burden of dengue. Nature.

[4] Castillo-Quino R, Vallejo-Castro E, Camacho-Aliaga AV, Canelas-Urey HI (2018). Adaptación del mosquito Aedes aegypti a 2 550 m s.n.m. Cochabamba, Bolivia, Febrero 2016. Gac Medica Boliv..

[5] Ruiz-López F, González-Mazo A, Vélez-Mira A, Gómez GF, Zuleta L, Uribe S (2016). Presencia de *Aedes* (Stegomyia) *aegypti* (Linnaeus, 1762) y su infección natural con el virus del dengue en alturas no registradas para Colombia. Biomedica.

[6] Castro A, Davila C, Laura W, Cubas F, Ávalos G, López Ocaña C, et al. Climas del Perú. Mapa de clasificación climática nacional. Servicio Nacional de Meteorología e Hidrología del Perú; 2020. https://idesep.senamhi.gob.pe/geonetwork/srv/spa/catalog.search#/metadata/9f18b911-%2064af-4e6b-bbef-272bb20195e4.

[7] Requena-Zúñiga E, Mendoza-Uribe L, Guevara-Saravia M (2016). New distribution areas of *Aedes aegypti* in Peru. Rev Peru Med Exp Salud Publica.

[8] Vera-Maloof FZ, Saavedra-Rodriguez K, Elizondo-Quiroga AE, Lozano-Fuentes S, Black WC (2015). Coevolution of the Ile 1,016 and Cys1, 534 mutations in the voltage gated sodium channel gene of *Aedes aegypti* in Mexico. PLoS Negl Trop Dis..

[9] Organización Mundial de la Salud. Respuesta Mundial para el Control de Vectores 2017-2030. 2017. http://www.who.int/malaria/areas/vector_control/Draft-WHO-GVCR-2017-2030-esp.pdf.

[10] Garcia-Rejon JE, Navarro JC, Cigarroa-Toledo N, Baak-Baak CM (2021). An updated review of the invasive *Aedes albopictus* in the Americas; geographical distribution, host feeding patterns, arbovirus infection, and the potential for vertical transmission of dengue virus. Insects..

[11] Andrade CS, Cáceres AG, Vaquerizo A, Ibañez-Bernal S, Cachay LS (2001). Reappearance of *Aedes aegypti* (Diptera: Culicidae) in Lima, Peru. Mem Inst Oswaldo Cruz.

[12] Phillips I, Need J, Escamilla J, Colán E, Sánchez S, Rodríguez M (1992). Dengue in the Peruvian Amazon. Bull PAHO.

[13] Mostorino ER, Rosas AA, Gutierrez PV, Anaya RE, Cobos ZM, García MM (2002). Manifestaciones clínicas y distribución geográfica de los serotipos del dengue en el Perú año 2001. Rev Peru Med Exp Salud Publica.

[14] Centro Nacional de Epidemiologia Prevención y Control de Enfermedades. Sala de situación de Salud—Perú a la SE 26-2021. 2021. https://www.dge.gob.pe/epipublic/uploads/asis-sala/asis-sala_202126.pdf.

[15] Mateo S (2017). Situación epidemiológica del Chikungunya en el Perú, SE 07–2017. Bol Epidemiol Peru.

[16] Mateo S (2017). Situación epidemiológica del Zika en el Perú, a la SE 07 2017. Bol Epidemiol Peru.

[17] Cabezas C, Fiestas V, García-Mendoza M, Palomino M, Mamani E, Donaires F (2015). Dengue in Peru: a quarter century after its reemergence. Rev Peru Med Exp Salud Publica.

[18] Chowell G, Cazelles B, Broutin H, Munayco CV (2011). The influence of geographic and climate factors on the timing of dengue epidemics in Perú, 1994–2008. BMC Infect Dis.

[19] Instituto Geográfico Nacional. Historia Del Instituto Geográfico Nacional. Primera ed. Enero. Lima; 2015. p. 195. https://cdn.www.gob.pe/uploads/document/file/2743191/LIBRO_IGN.pdf.pdf.

[20] Ministerio de salud. Infestación por *Aedes aegypti1 1*. 2021 [cited 02 Aug 2021] http://www.digesa.minsa.gob.pe/DCOVI/infestacion.pdf.

[21] Bueno C, Vela F, Llontop A, Carranza J. Dengue en San Martín: Seis Años de Experiencias. Dirección Regional de Salud San Martín; 1998. p. 61. https://cdn.www.gob.pe/uploads/document/file/392260/Dengue_en_San_Martin__Seis_años_de_experiencias20191017-26355-19awao1.pdf.

[22] Palomino M (2018). Vigilancia de la resistencia a los insecticidas Enero—Junio 2019. Bol Inst Nac Salud.

[23] Pinto J, Palomino M, Mendoza-Uribe L, Sinti C, Liebman KA, Lenhart A (2019). Susceptibility to insecticides and resistance mechanisms in three populations of *Aedes aegypti* from Peru. Parasit Vectors.

[24] Organización Panamericana de la Salud (2019). Orientaciones para la estructuración de laboratorios de entomología en salud pública.

[25] Organización Mundial de la Salud (OMS). Directrices para el control de vectores del paludismo [Guidelines for malaria vector control]. Ginebra: Organización Mundial de la Salud; Licencia: CC BY-NC- SA 3.0 IGO.; 2019. https://apps.who.int/iris/bitstream/handle/10665/330723/9789243550497-spa.pdf?ua=1.

[26] Organización Panamericana de la Salud. Documento operativo de aplicación del manejo integrado de vectores adaptado al contexto de las Américas. Documento operativo de aplicación del manejo integrado de vectores adaptado al contexto de las Américas. Washington DC: OPS; 2019. https://iris.paho.org/handle/10665.2/51760.

[27] Palomino M. Protocolo sanitario de urgencia para el reforzamiento de la vigilancia entomológica del vector *Aedes aegypti* mediante el uso de ovitrampas para establecimientos de salud. Vol. 1. R.M. No. 010–2015. Lima: MINSA; 2016. http://bvs.minsa.gob.pe/php/index.php.

[28] Georghiou GP, Wirth M, Tran H, Saume F, Knudsen AB (1987). Potential for organophosphate resistance in *Aedes aegypti* (Diptera: Culicidae) in the Carribean area and neighboring countries. J Med Entomol.

[29] Reiter P, Amador MA, Colon N (1991). Enhancement of the CDC ovitrap with hay infusions for daily monitoring of *Aedes aegypti* populations. J Am Mosq Control Assoc.

[30] Consoli RAGB, Oliveira RL de. Principais mosquitos de importância sanitária no Brasil. Principais mosquitos de importância sanitária no Brasil. Rio de Janeiro: Editora FIOCRUZ; 1994. http://books.scielo.org/id/th.

[31] Da-Cunha MP, Lima JBP, Brogdon WG, Moya GE, Valle D (2005). Monitoring of resistance to the pyrethroid cypermethrin in Brazilian *Aedes aegypti* (Diptera Culicidae) populations collected between 2001 and 2003. Mem Inst Oswaldo Cruz.

[32] WHO. Monitoring and managing insecticide resistance in* Aedes mosquito* populations: interim guidance for entomologists. 2016. https://apps.who.int/iris/handle/10665/204588.

[33] de Sá ELR, Rodovalho CDM, de Sousa NPR, de Sá ILR, Bellinato DF, Dias LDS (2019). Evaluation of insecticide resistance in *Aedes aegypti* populations connected by roads and rivers: the case of Tocantins state in Brazil. Mem Inst Oswaldo Cruz.

[34] Raymond M (1985). Presentation d’une programme d’analyse logprobit pour microordinateur cahiers Orstrom. Sér Ent Med Parasitol.

[35] Mazzarri MB, Georghiou GP (1995). Characterization of resistance to organophosphate, carbamate, and pyrethroid insecticides in field populations of *Aedes aegypti* from Venezuela. J Am Mosq Control Assoc.

[36] Centro Nacional de Epidemiología P y C. Reporte de Situación epidemiológica a nivel regional y distrital. Lima: Ministerio de Salud del Perú. [cited 10 Apr 2022] https://www.dge.gob.pe/salasituacional/sala/index/SalaRegional/145.

[37] CDC-MINSA-Perú. Sala de Situación de Salud: Perú a la SE 52—2021. Ministerio de Salud del Perú. 2021. https://www.dge.gob.pe/epipublic/uploads/asis-sala/asis-sala_202152_14_073134.pdf.

[38] Bustios C, Rios A, Arroyo R, Marquez C, Miano J. La malaria y el dengue en la historia de la salud pública peruana 1821–2011. 2014. http://bvs.minsa.gob.pe/local/MINSA/3425.pdf.

[39] Valle J. Reinfestación de la selva peruana por *Aedes aegypti* Linneo, 1762 (Diptera:Culicidae). Thesis. Lima: Universidad Ricardo Palma; 1989.

[40] Morales D, Ponce P, Cevallos V, Espinosa P, Vaca D, Quezada W (2019). Resistance status of *Aedes aegypti* to deltamethrin, malathion, and temephos in Ecuador. J Am Mosq Control Assoc.

[41] Neyra D, Cabezas C, Ruebush T (2003). El proceso de adecuación y cambio en la política del tratamiento de la malaria por *Plasmodium falciparum* en el Perú, 1990–2001. Rev Peru Med Exp Salud Publica.

[42] Organización Panamericana de la Salud (OPS) (2000). No 8 Crónicas de desastres Fenómeno El Niño, 1997–1998.

[43] Organización Panamericana de la Salud (OPS) (2019). Abordaje de los determinantes ambientales de la salud en las estrategias de vigilancia y control de vectores: orientaciones para promover intervenciones clave.

[44] Valdez W, Ramos W, Miranda J, Tovar JC. Análisis de la situación de salud del Perú. Primera Ed. Lima: Ministerio de Salud; 2010. https://www.researchgate.net/publication/283429657_Analisis_de_la_situacion_de_Salud_del_Peru_2010.

[45] Fernández WF, Iannacone J (2005). Variaciones de tres índices larvarios de *Aedes aegypti* (L.) (Diptera: Culicidae) y su relación con los casos de dengue en Yurimaguas, Perú, 2000–2002. Parasitol Latinoam..

[46] Barrantes R, Glave M, editores. Amazonía peruana y desarrollo económico. Primera Ed. Lima: Grupo de Análisis para el Desarrollo (GRADE), Instituto de Estudios Peruanos (IEP); 2014. https://repositorio.iep.org.pe/bitstream/handle/IEP/601/estudiossobredesigualdad8.pdf?sequence=2&isAllowed=y.

[47] Ministerio de Salud. Aprendiendo de la experiencia. Lecciones aprendidas para la preparación y respuesta en el control vectorial ante brotes de dengue en el Perú. Lima: Ministerio de Salud, Dirección General de Salud Ambiental; 2011. http://bvs.minsa.gob.pe/local/MINSA/1828.pdf.

[48] Dirección de Epidemiología. Análisis de situación de salud 2016. Madre de Dios: Dirección Regional de Salud Madre de Dios; 2016. http://dge.gob.pe/portal/Asis/indreg/asis_madrededios.pdf.

[49] Salmón-Mulanovich G, Blazes DL, Guezala VMC, Rios Z, Espinoza A, Guevara C (2018). Individual and spatial risk of dengue virus infection in Puerto Maldonado, Peru. Am J Trop Med Hyg.

[50] Chediak M, Pimenta FG, Coelho GE, Braga IA, Lima JBP, Cavalcante KRLJ (2016). Spatial and temporal country-wide survey of temephos resistance in Brazilian populations of *Aedes aegypti*. Mem Inst Oswaldo Cruz.

[51] López Rodríguez R. Estudio de la sensibilidad y/o resistencia a los insecticidas del *Aedes aegypti*, vector del dengue en Bolivia. Barcelona: Universidad de Barcelona; 2015. https://repositorio.umsa.bo/xmlui/bitstream/handle/123456789/10459/TMT040.pdf?sequence=5&isAllowed=y.

[52] Montoro Y, Moreno R, Gomero L, Reyes M (2009). Características de uso de plaguicidas químicos y riesgos para la salud en agricultores de la Sierra Central del Perú. Rev Peru Med Exp Salud Publica.

[53] Tupayachi Calderón ER. Transferencia de tecnología para el uso adecuado de plaguícidas agrícolas. Lima: Universidad Nacional Agraria La Molina; 2020. https://hdl.handle.net/20.500.12996/4344.

[54] Guerrero-Padilla AM, Otiniano-Medina LJ (2012). Impacto en agroecosistemas generado por pesticidas en los sectores Vichanzao, El Moro, Santa Lucía de Moche y Mochica Alta, Valle de Santa Catalina, La Libertad. Perú Sciéndo.

[55] Consejo Nacional del Ambiente, Dirección General de Salud Ambiental, Servicio Nacional de Sanidad Agraria. Plan nacional de implementación del convenio de Estocolmo sobre los contaminantes orgánicos persistentes en el Perú. 1st Ed. 2007. https://sinia.minam.gob.pe/download/file/fid/39108.

[56] Thongwat D, Bunchu N (2015). Susceptibility to temephos, permethrin and deltamethrin of *Aedes aegypti* (Diptera: Culicidae) from Muang district, Phitsanulok Province. Thailand. Asian Pac J Trop Med..

[57] Wirth MC, Georghiou GP (1999). Selection and characterization of temephos resistance in a population of *Aedes aegypti* from Tortola, British Virgin Islands. J Am Mosq Control Assoc.

[58] Moyes CL, Vontas J, Martins AJ, Ng LC, Koou SY, Dusfour I (2017). Contemporary status of insecticide resistance in the major *Aedes* vectors of arboviruses infecting humans. PLoS Negl Trop Dis.

[59] Muthusamy R, Shivakumar MS (2015). Susceptibility status of *Aedes aegypti* (L.) (Diptera: Culicidae) to temephos from three districts of Tamil Nadu, India. J Vector Borne Dis..

[60] Conde M, Orjuela LI, Castellanos CA, Herrera-Varela M, Licastro S, Quiñones ML (2015). Evaluación de la sensibilidad en poblaciones de *Aedes aegypti* (Diptera: Culicidae) del departamento de Caldas, Colombia, en 2007 y 2011. Biomedica.

[61] Araújo AP, Araujo Diniz DF, Helvecio E, de Barros RA, de Oliveira CMF, Ayres CFJ (2013). The susceptibility of *Aedes aegypti* populations displaying temephos resistance to Bacillus thuringiensis israelensis: a basis for management. Parasit Vectors.

[62] Marcombe S, Paris M, Paupy C, Bringuier C, Yebakima A, Chandre F (2013). Insecticide-driven patterns of genetic variation in the dengue vector *Aedes aegypti* in Martinique Island. PLoS ONE.

[63] Andrighetti MTM, Cerone F, Rigueti M, Galvani KC, Da Graça Macoris ML (2008). Effect of pyriproxyfen in *Aedes aegypti* populations with different levels of susceptibility to the organophosphate temephos. Dengue Bull.

[64] Marcombe S, Fustec B, Cattel J, Chonephetsarath S, Thammavong P, Phommavanh N (2019). Distribution of insecticide resistance and mechanisms involved in the arbovirus vector *Aedes aegypti* in Laos and implication for vector control. PLoS Negl Trop Dis.

[65] Aguirre-Obando OA, Pietrobon AJ, Bona ACD, Navarro-Silva MA (2016). Contrasting patterns of insecticide resistance and knockdown resistance (kdr) in *Aedes aegypti* populations from Jacarezinho (Brazil) after a Dengue Outbreak. Rev Bras Entomol.

[66] Aguirre-Obando OA, Bona ACD, Duque LJE, Navarro-Silva MA (2015). Insecticide resistance and genetic variability in natural populations of *Aedes* (Stegomyia) *aegypti* (Diptera: Culicidae) from Colombia. Zoologia.

[67] Tikar SN, Mendki MJ, Chandel K, Parashar BD, Prakash S (2008). Susceptibility of immature stages of *Aedes* (Stegomyia) *aegypti*; vector of dengue and chikungunya to insecticides from India. Parasitol Res.

[68] Macoris MDLDG, Andrighetti MTM, Otrera VCG, de Carvalho LR, Caldas Júnior AL, Brogdon WG (2007). Association of insecticide use and alteration on *Aedes aegypti* susceptibility status. Mem Inst Oswaldo Cruz.

[69] Ishak IH, Jaal Z, Ranson H, Wondji CS (2015). Contrasting patterns of insecticide resistance and knockdown resistance (kdr) in the dengue vectors *Aedes aegypti* and *Aedes albopictus* from Malaysia. Parasit Vectors.

[70] Rocha HDR, Paiva MHS, Silva NM, de Araújo AP, dos Reis da Rosa de Azevedo Camacho D, da Moura AJF (2015). Susceptibility profile of *Aedes aegypti* from Santiago Island, Cabo Verde, to insecticides. Acta Trop.

[71] Ministerio de Salud. Boletin Epidemiologico Del Peru 2016 SE.52. 1st ed. Bueno C, Lizarbe M, Cruz A, ed. Lima: Ministerio de Salud; 2017. https://www.dge.gob.pe/portal/docs/vigilancia/boletines/2016/52.pdf.

[72] Rawlins SC, Wan JO (1995). Resistance in some Caribbean populations of *Aedes aegypti* to several insecticides. J Am Mosq Control Assoc.

[73] Rawlins SC (1998). Spatial distribution of insecticide resistance in Caribbean populations of *Aedes aegypti* and its significance. Rev Panam Salud Pública.

[74] Lima EP, Paiva MHS, de Araújo AP, da Silva EVG, da Silva UM, de Oliveira LN (2011). Insecticide resistance in *Aedes aegypti* populations from Ceará, Brazil. Parasit Vectors.

[75] Rahman RU, Cosme LV, Costa MM, Carrara L, Lima JBP, Martins AJ (2021). Insecticide resistance and genetic structure of *Aedes aegypti* populations from rio de Janeiro state, Brazil. PLoS Negl Trop Dis.

[76] Rodríguez MM, Bisset JA, Fernández D (2007). Levels of insecticide resistance and resistance mechanisms in *Aedes aegypti* from some Latin American countries. J Am Mosq Control Assoc.

