# Supplementary material for: First national-scale evaluation of temephos resistance in Aedes aegypti in Peru
Source: Parasit Vectors. 2022 Jul 11;15:254. doi: 10.1186/s13071-022-05310-x (PMC9397858; doi:10.1186/s13071-022-05310-x)
Supplement: Supplementary file 1 — Additional file 1: Table S1. Number of cases of dengue according to departments and geographic regions, Peru 2015–2021. [file 13071_2022_5310_MOESM1_ESM.docx]

Table S1. Number of cases of dengue according to departments and geographic regions, Peru 2015 - 2021.

Table prepared with data provided by the Centro Nacional de Epidemiología, Prevención y Control Enfemerdades (CDC-Ministerio de Salud, Peru). <https://www.dge.gob.pe/portalnuevo/publicaciones/salas-de-situacion-semanal/>
